# Accuracy of the InnowaveDX MTB/RIF test for detection of *Mycobacterium tuberculosis* and rifampicin resistance: a prospective multicentre study

**DOI:** 10.1080/22221751.2022.2151382

**Published:** 2023-01-02

**Authors:** Yunfeng Deng, Zichun Ma, Biyi Su, Guanghong Bai, Jianhua Pan, Quan Wang, Long Cai, Yanhua Song, Yuanyuan Shang, Pinyun Ma, Jing Li, Qianxuan Zhou, Gulibike Mulati, Dapeng Fan, Shanshan Li, Yaoju Tan, Yu Pang

**Affiliations:** a Katharine Hsu International Research Center of Human Infectious Diseases, Shandong Public Health Clinical Center Affiliated to Shandong University, Jinan, People’s Republic of China; b Department of Bacteriology and Immunology, Beijing Key Laboratory on Drug-Resistant Tuberculosis Research, Beijing Chest Hospital, Capital Medical University, Beijing Tuberculosis & Thoracic Tumor Research Institute, Beijing, People’s Republic of China; c Department of Clinical Laboratory, Guangzhou Chest Hospital, Guangzhou, People’s Republic of China; d Department of Clinical Laboratory, Shanxi Provincial Tuberculosis Hospital, Xi’an, People’s Republic of China; e Department of Clinical Laboratory, Changsha Central Hospital, Changsha, People’s Republic of China; f Department of Clinical Laboratory, The Eighth Affiliated Hospital of Xinjiang Medical University, Urumqi, People’s Republic of China; g Department of Clinical Laboratory, Hangzhou Red Cross Hospital, Hangzhou, People’s Republic of China; h Department of Tuberculosis, Beijing Key Laboratory on Drug-Resistant Tuberculosis Research, Beijing Chest Hospital, Capital Medical University, Beijing Tuberculosis & Thoracic Tumor Research Institute, Beijing, People’s Republic of China

**Keywords:** Tuberculosis, molecular diagnosis, *rpoB*, rifampicin, GeneXpert, InnowaveDX

## Abstract

Early and accurate diagnosis of tuberculosis (TB) is necessary to initiate proper therapy for the benefit of the patients and to prevent disease transmission in the community. In this study, we developed the InnowaveDX MTB/RIF (InnowaveDX) to detect *Mycobacterium tuberculosis* (MTB) and rifampicin resistance simultaneously. A prospective multicentre study was conducted to evaluate the diagnostic performance of InnowaveDX for the detection MTB in sputum samples as compared with Xpert and culture. The calculated limit of detection (LOD) for InnowaveDX was 9.6 CFU/ml for TB detection and 374.9 CFU/ml for RIF susceptibility. None of the other bacteria tested produced signals that fulfilled the positive TB criteria, demonstrating a species-specificity of InnowaveDX. Then 951 individuals were enrolled at 7 hospitals, of which 607 were definite TB cases with positive culture and/or Xpert results, including 354 smear-positive and 253 smear-negative cases. InnowaveDX sensitivity was 92.7% versus bacteriologically TB standard. Further follow-up revealed that 61 (91.0%) out of 67 false-positive patients with no bacteriological evidence met the criteria of clinically diagnosed TB. Among 125 RIF-resistant TB patients diagnosed by Xpert, 108 cases were correctly identified by InnowaveDX, yielding a sensitivity of 86.4%. Additionally, the proportion of very low bacterial load in the discordant susceptibility group was significantly higher than in the concordant susceptibility group (*P* = 0.029). To conclude, we have developed a novel molecular diagnostic with promising detection capabilities of TB and RIF susceptibility. In addition, the discordant RIF susceptibility results between InnowaveDX and Xpert are more frequently observed in samples with very low bacterial load.

## Introduction

Tuberculosis (TB) remains a major global public health priority [1,2]. The World Health Organization (WHO) estimated that 9.9 million people developed active TB, with 1.5 million deaths from TB in 2020 [2]. An alarming increase of drug-resistant *Mycobacterium tuberculosi*s (MTB) infections exacerbates the TB epidemic, and leads to the emergence of resistance to additional antituberculosis drugs [3,4]. Smear microscopy is the most widely used test for the diagnosis of TB, but its poor sensitivity and incapability to distinguish nontuberculous mycobacteria from MTB creates a diagnostic challenge [5]. Mycobacterial culture yields promising sensitivity to detect MTB from multiple specimens; however, the long turn-around time leads to delay in reporting results [6,7]. Thus, novel assays that are suitable for early and accurate diagnosis of TB and drug-resistant TB is necessary to initiate proper therapy for the benefit of the patient and to prevent disease transmission in the community.

Advances in molecular techniques have greatly increased our understanding of tubercle bacilli, and has identified specific molecular targets used for detection of this pathogen, as well as prediction of drug resistance [8]. More recently, many high burden countries have scaled-up rapid molecular tests for the diagnosed of TB, particularly Xpert MTB/RIF (Cepheid, Sunnyvale, CA), an automated, real-time nucleic acid amplification assay that simultaneously detects the presence of MTB and resistance to rifampicin [9,10]. The WHO has endorsed the routine use of this assay as the initial test for patients with symptoms suggestive of TB to accelerate TB diagnosis [11]; however, the relative high cost and long processing intervals constitutes a hurdle for resource-constrained settings to widespread use of Xpert as a point-of-care (POC) test [12]. There need to be more efforts to develop molecular diagnostics that can yield high sensitivity in a POC manner.

Recently, The InnowaveDX MTB/RIF (InnowaveDX), a novel real-time PCR assay for MTB that is able to detect rifampicin resistance simultaneously, was developed by InnowaveDX company (Suzhou, China). It integrates sample processing and detection in a single-use cartridge. The PCR assay amplifies partial IS6110 and rpoB segments in a real-time PCR. Seven fluorescent probes are designed to detect target sequences, including five overlapping fluorescent probes A, B, C, D and E, targeting the 81-bp rifampin resistance determining region (RRDR) of *rpoB* gene, one probe targeting the conserved region of *rpoB* gene outside RRDR as internal control for real-time amplification assay of *rpoB*, and one probe targeting IS6110 element. The combined detection of *rpoB* and IS6110 enables the assay to detect paucibacillary samples. We hypothesized that InnowaveDX MTB/RIF would have improved sensitivity in comparison to Xpert MTB/RIF. In the present study, we conducted a prospective multicentre study to evaluate the diagnostic performance of InnowaveDX for detection MTB in sputum samples as compared with Xpert and culture. Additionally, it might be helpful to clearly mention the technical difference between the novel assay and the GeneXpert Ultra assay.

## Methods

### Analytical sensitivity and specificity of InnowaveDX

The analytical sensitivity and limit of detection (LOD) of MTB was performed according to a standard protocol as previously described [13]. Briefly, TB assay was performed by spiking reference MTB H37Rv (ATCC27294) into MTB-naïve sputum at a series of concentrations (1.56 to 50 CFU/ml for TB assay and 62.5 CFU/ml to 2 × 10^3^ CFU/ml for RIF susceptibility analysis). Each sample with a defined dilution was tested 20 times. The assay LOD was defined as the lowest number of CFU which would yield the detection of MTB ≥95% of the time that a test was performed [13]. In addition, the analytical specificity of InnowaveDX was tested using concentrated bacterial cultures (approximately 10^8^ CFU/ml) of 14 different species of nontuberculous mycobacteria (NTM) and other bacteria (Table S1).

### Study population

In our prospective multicentre study, we enrolled adults with symptoms suggestive of active TB between September 2021 and February 2022 in seven TB-specialized hospitals in China. The seven TB-specialized hospitals were as follows: Beijing Chest Hospital, Guangzhou Chest Hospital, Shandong Public Health Clinical Center, Hangzhou Red Cross Hospital, Shanxi Provincial Tuberculosis Institute, Changsha Central Hospital, and the Eighth Affiliated Hospital of Xinjiang Medical University. The patients included in this study met the following eligibility criteria: (i) the age older than 18 years; (ii) patients with at least 2 weeks of cough; (iii) patients providing qualified sputum samples. After enrolment, each patient was requested to produce at least of 5 ml of sputum, which was used for laboratory examinations. The demographic and clinical characteristics were collected by using standardized e-form. Patients were excluded from the analysis if culture contamination did not allow further analysis or if results of Xpert or InnowaveDX were indeterminate. As per standard clinic guidelines, patients had chest radiography and sputum sampling after enrolment, and were scheduled to return to outpatient care for clinical assessment. The pulmonary TB patients were diagnosed and classified into one of two diagnostic categories following the Diagnosis for Pulmonary Tuberculosis (WS288-2017) Guidelines: (i) confirmed TB patients: clinical TB symptoms with at least one sputum sample culture- and/or Xpert-positive for MTB, which had bacteriological laboratory evidence for Mtb infection; (ii) clinically diagnosed TB patients: clinical TB symptoms without any positive laboratory evidence by culture and Xpert plus clinical positive response to anti-TB treatment. The study was approved by the Ethics Committee of Shandong Public Health Clinical Center (2021XKYYEC-11). Written informed consent was given from each patient prior to the enrolment.

### Procedures

Sputum samples were sent for processing at the Clinical Laboratory at each site. A loopful of sputum was prepared, stained with Auramine O method, and graded according to standard national guideline [14]. Then sputum was processed with N-acetyl-L-cysteine and sodium hydroxide and centrifuged. After washing with phosphate buffer saline (PBS), the pellets were resuspended in 2.5 mL PBS. 0.5 mL of the sediment was inoculated into a Mycobacterial Growth Indicator Tube (MGIT, Becton Dickinson Diagnostics; Franklin Lakes, NJ) and incubated for no more than 6 weeks. MGIT-positive cultures were confirmed for the presence of MTB by Capilia TB MPT64 Rapid test (Genesis, Hangzhou, China). The *in vitro* drug susceptibility including rifampicin (RIF), isoniazid (INH), ethambutol (EMB) and pyrazinamide (PZA) was determined for all positive cultures using the phenotypic drug susceptibility testing method endorsed by the World Health Organization.

For the Xpert MTB/RIF assay, the 1.0 mL of raw sputum was added to 2.0 mL of the sample reagent according to the manufacturer's instructions. After incubation for 15 min, 2.0 mL of this mixture was pipetted to the Xpert MTB/RIF assay cartridge and the cartridge was loaded in a GeneXpert instrument. The Xpert assays provides semi-quantitative results based on the cycle threshold of the first positive *rpoB* probe, including high, medium, low and very low.

For the InnowaveDX assay, the 1.0 mL of raw sputum was pipetted to 6.0 mL of the lysis buffer containing 150 μL internal reference for control purpose. The tube containing the sample was then proceeded with ultrasonic treatment and incubated at room temperature for 15 min. The DNA extraction were automatically performed using magnetic bead method, in which small magnetic particles were used to capture nucleic acids. Then the preparation of PCR mixture and DNA amplification were also completed in the InnowaveDX instrument within 2 h automatically. The results were interpreted by pre-installed software, including the presence of tubercle bacilli and genotype variations within *rpoB* locus. The software judged samples with >4 cycles difference in Cycle Threshold (Ct) values between any probe targeting RRDR and internal control probe of *rpoB* gene as resistance to RIF.

### Statistical analysis

Published data suggested that 50% of the study population would have a positive culture and/or Xpert result. At this prevalence, a total sample size of more than 430 participants was required to establish whether the InnowaveDX assay could achieve an estimated sensitivity of 85% and an estimated specificity of 95% with a 5% margin of error. We calculated the sensitivity of InnowaveDX for detection of MTB using microbiological or clinical reference standard, respectively. The microbiological reference standard was defined as patients with at least one sputum sample culture and/or Xpert-positive for MTB; whereas the clinical reference standard is defined as patients with final diagnosis of pulmonary TB, including confirmed and clinically diagnosed TB patients. For RIF susceptibility, its sensitivity was assessed using Xpert as reference standard. Analyses of the diagnostic accuracy of the InnowaveDX test and comparator tests were performed per case and described as point estimates and 95% confidence intervals. The consistency of InnowaveDX and Xpert in detecting RIF susceptibility was conducted with unweighted Kappa analysis. The comparisons in diagnostic accuracy across different tests were conducted with Chi-square test. A *P* value less than 0.05 indicates significant difference. All calculations were performed using SPSS version 20.0 (IBM, Chicago, IL).

## Results

### LOD and analytical specificity of InnowaveDX

**Figure 1.**
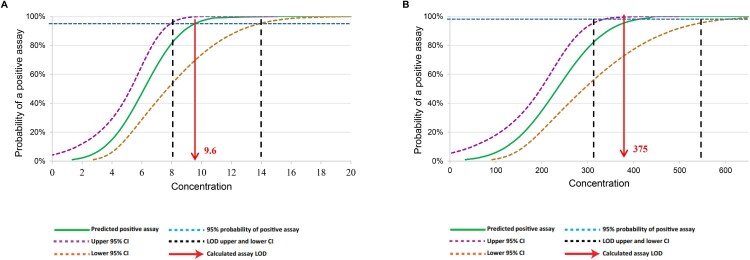
Limit of detection for the reference MTB H37Rv. The limit of detection of tuberculosis detection is shown for TB detection (A) and rifampicin susceptibility (B).

We firstly tested the LOD with clinical sputum samples spiked with known number of tubercle bacilli. Analytical tests revealed that InnowaveDX could detect the presence of MTB correctly 100% of the time that a sample was tested, down to dilutions to 12.5 CFU/ml. The rates of correct TB-positive specimen detection by Xpert decreased to 10% (2/20) and 50% (10/20) for samples with concentrations at 3.1 and 6.2 CFU/ml, respectively. The calculated TB detection LOD for InnowaveDX was 9.6 CFU/ml (95% confidence interval [CI], 7.9–13.9) (Figure 1). In addition, correct detection of RIF susceptibility by InnowaveDX was 100% up to 500 CFU/ml; whereas 55% of the samples tested were correctly identified at 250 CFU/ml, resulting in a calculated LOD of 374.9 CFU/ml (95% CI, 309.9–538.3 CFU/ml) for RIF susceptibility. Thus, its limit of detection for the presence of MTB in sputum appeared to be close to that of Xpert MTB/RIF Ultra and at least one order of magnitude more sensitive than the mycobacterial culture and Xpert MTB/RIF. We investigated analytical specificity using sputum containing 106 CFU/ml of 20 NTM species, including 14 different species of nontuberculous mycobacteria (NTM) and other bacteria. None of the bacteria tested produced signals that fulfilled the positive TB criteria, demonstrating a species-specificity of fluorescence probes.

### Study participants and diagnostic performance for TB

**Figure 2.**
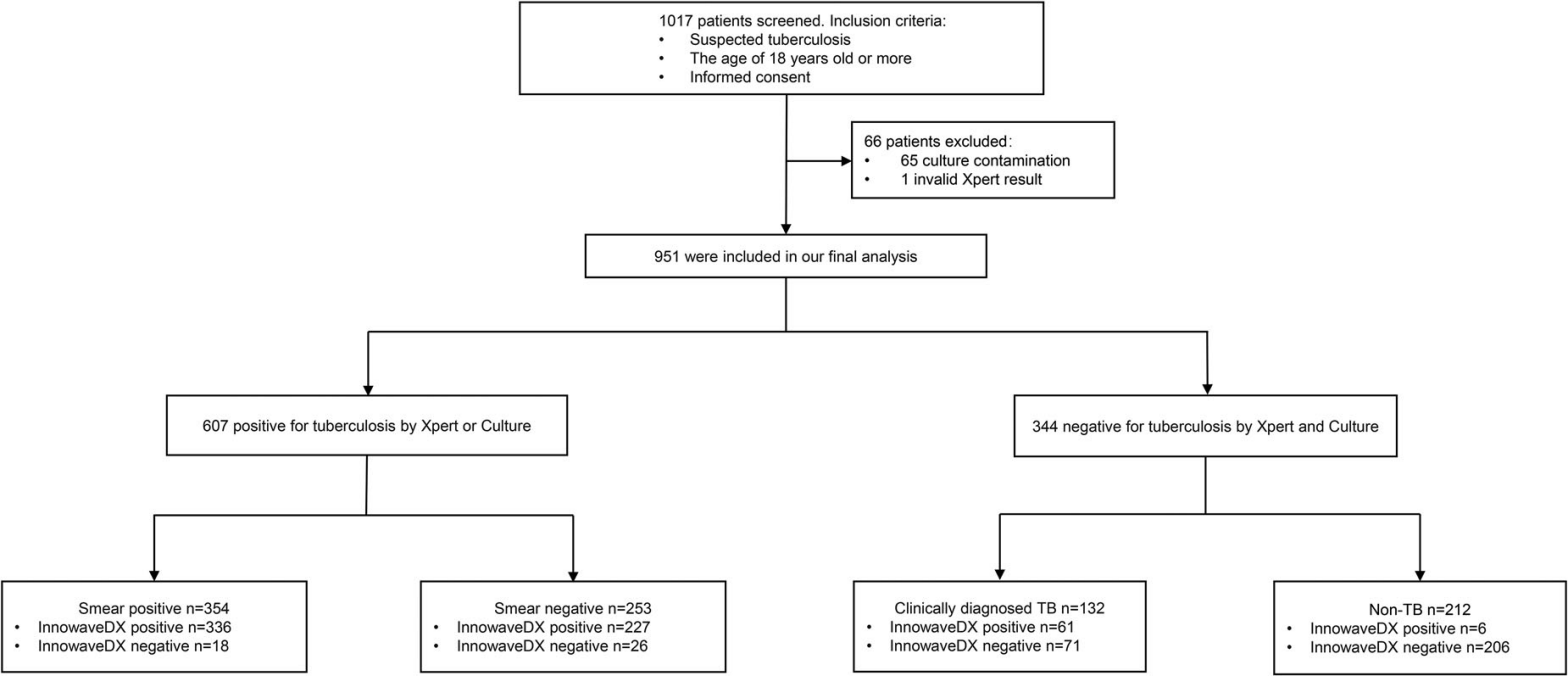
Participant enrolment.

In total, 1017 individuals were initially enrolled at the 7 sites. Subsequently, 66 individuals were excluded from the analysis due to invalid Xpert results or culture contamination. Thus, the final sample size used for our analysis were 951 patients, which included 739 (77.7%) with active TB (607 bacteriologically confirmed cases and 132 clinically diagnosed cases) and 212 (22.3%) without TB (Figure 2). The demographic characteristics of these patients were summarized in Table S2. InnowaveDX sensitivity was 92.7% (563/607; 95% CI: 90.3%-94.6%) versus bacteriologically TB standard. When stratified by the result of sputum smear, the sensitivity was 94.9% (336/354; 95%CI: 91.9%-96.9%) and 89.7% (227/253; 95%CI: 85.1%-93.0%) for smear-positive and smear-negative patients, respectively. Against this reference standard, the specificity of InnowaveDX was 80.5% (277/344; 95%CI: 75.9%–84.5%), of whom 67 patients with no laboratory evidence had positive results by the InnowaveDX assay. Additionally, the positive predictive value (PPV) and negative predictive value (NPV) of this assay was 89.3% (95% CI: 86.6%–91.6%) and 86.3% (95% CI: 81.9%–89.8%), respectively. Further follow-up revealed that 61 (91.0%) out of 67 false positive patients met the criteria of clinically diagnosed TB; whereas the remaining 6 (9.0%) patients had no definite clinical diagnosis (Table 1). Using clinical reference standard, the sensitivity and specificity of InnowaveDX assay were 84.4 (624/739; 95%CI: 81.6%–86.9%) and 97.2 (206/212; 95%CI: 93.7%–98.8%), respectively (Table 2).

**Table 1.** Diagnostic performance of the InnowaveDX assay for TB using microbiological reference standard.

Reference	Sensitivity (%, 95%CI)			Specificity (%, 95%CI)	PPV (%, 95% CI)	NPV (%, 95% CI)
	All	Smear positive	Smear negative			
Microbiological reference standard^a^	563/607 92.7 (90.3–94.6)	336/354 94.9 (91.9–96.9)	227/253 89.7 (85.1–93.0)	277/344 80.5 (75.9–84.5)	563/630 89.3 (86.6–91.6)	277/321 86.3 (81.9–89.8)

^a^ The microbiological reference standard is defined as patients with at least one sputum sample culture- and/or Xpert-positive for MTB.

PPV: positive predictive value; NPV: negative predictive value; CI: confidential interval; TB: tuberculosis; MTB: *Mycobacterium tuberculosis*.

**Table 2.** Diagnostic performance of the InnowaveDX assay for TB using clinical reference standard.

Reference	Sensitivity (%, 95% CI)	Specificity (%, 95% CI)	PPV (%, 95% CI)	NPV (%, 95% CI)
Clinical reference standard^a^	624/739 84.4 (81.6–86.9)	206/212 97.2 (93.7–98.8)	624/630 99.0 (97.8–99.6)	206/321 64.2 (58.6–69.4)

^a^ The clinical reference standard is defined as patients with final diagnosis of pulmonary TB.

PPV: positive predictive value; NPV: negative predictive value; CI: confidential interval.

**Figure 3.**
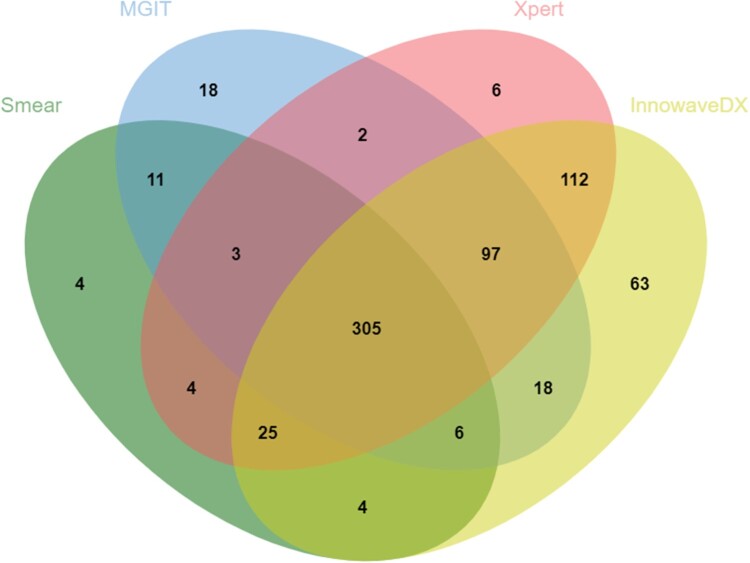
Venn diagram of overlap in MTB detection using InnowaveDX, Xpert, smear and mycobacterial MGIT culture in sputum sample. Abbreviations: MGIT, mycobacterial growth indicator tube.

Considering the distribution and overlap of positive specimen by Xpert, MGIT and InnowaveDX, the majority of positive Xpert (539/554), and MGIT cases (426/460) were also positive by InnowaveDX. In addition, 6 and 63 positive patients were solely reported by Xpert and InnowaveDX, respectively (Figure 3). Of all definite and clinically diagnosed TB patients, the proportion of MTB detected by InnowaveDX was 84.4% (624/739), which was significantly higher than results for Xpert (75.0%, 554/739, *P *< 0.01) (Figure 3). When combined use of Xpert and InnowaveDX, this proportion was 87.3% (645/739), which was also significantly higher than the sole use of Xpert test (*P *< 0.01).

## Performance of the InnowaveDX assay for RIF susceptibility

**Figure 4.**
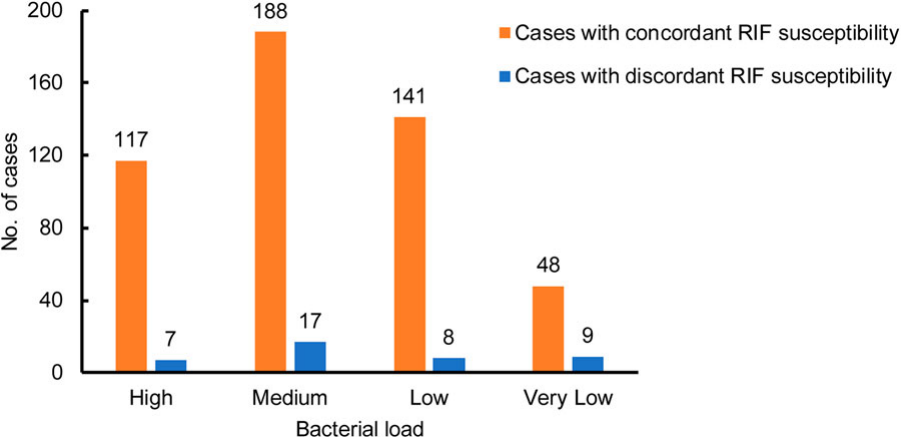
Distribution of cases with discordant RIF susceptibility between Xpert and InnowaveDX stratified by bacterial load.

We evaluated the diagnostic accuracy of the InnowaveDX assay versus Xpert for detection of RIF resistance. As shown in Table 2, among 125 RIF-resistant TB patients diagnosed by Xpert, 108 cases were correctly identified by InnowaveDX, yielding a sensitivity of 86.4% (95% CI: 78.8%–91.6%). Additionally, 386 out of 410 RIF-susceptible patients diagnosed by Xpert were confirmed by InnowaveDX, demonstrating a specificity of 94.1% (95% CI: 91.3%–96.1%) (Table 3). Kappa analysis was used to compare the consistency of InnowaveDX and Xpert in detecting RIF susceptibility and the results showed a Kappa value of 0.79, indicating an overall comparable diagnostic accuracy. Of the 535 cases we evaluated, 41 cases presented inconsistent RIF susceptibility results between InnowaveDX and Xpert. We analysed the results of these isolates, while 11 out of 41 were culture negative. Finally, we reviewed *in vitro* DST results of these 30 isolates, of which 19 (63.3%) were consistent with the Xpert results, the other 11 (36.7%) were consistent with the InnowaveDX results. When the distribution of discordant cases was also stratified based on different bacterial load after analysis using Xpert, comparisons made between the two groups showed that the proportion of very low bacterial load in the discordant group (9/41, 22.0%) was significantly higher than in the concordant group (48/494, 9.7%, *P *= 0.029), indicating a major contribution of bacterial load to discordant results by molecular diagnostics (Figure 4).

**Table 3.** Diagnostic accuracy of the InnowaveDX assay for RIF susceptibility against Xpert.

InnowaveDX	Xpert		Total	Sensitivity (%, 95% CI)	Specificity (%, 95% CI)	PPV (%, 95% CI)	NPV (%, 95% CI)
	R	S					
R	108	24	132	86.4 (78.8–91.6)	94.1 (91.3–96.1)	81.8 (74.0–87.8)	95.8 (93.2–97.4)
S	17	386	403				
Total	125	410	535				

RIF: rifampicin; R: resistant; S: susceptible; PPV: positive predictive value; NPV: negative predictive value; CI: confidential interval.

## Discussion

Novel diagnostics with improved sensitivity are urgently required to assist diagnosis and guide treatment decision for pulmonary TB [15]. In the study, we have developed a novel molecular diagnostic, namely InnowaveDX, with promising detection capabilities of TB and RIF susceptibility. Analyses using sputum spiked with known numbers of MTB CFU predicted a clinical LOD of 9.6 CFU/ml, which was 10-fold increase in analytical sensitivity for the detection of H37Rv compared to Xpert (131 CFU/ml) [16], but comparable to results for Xpert Ultra (15.6 CFU/ml) [13]. Several procedures serve as plausible explanations for the remarkable increase in detection sensitivity. On the one hand, the InnowaveDX employs two detection targets, including *rpoB* and multicopy IS6110 [13]. Undoubtedly, the use of the multicopy gene improves the sensitivity of the assay over a single copy gene. On the other hand, an optimal nucleic acid sample preparation is of great importance for the sensitivity of nucleic acid amplification technologies [17]. However, mycobacteria cells are strongly resistance to conventional lysis methods due to their markedly thickened cell wall [18], and often requires a combination of techniques to enhance the effectiveness of cell lysis [17]. Previous experimental studies revealed that the integration of ultrasound technology into DNA purification procedures could significantly disrupt cell wall structure and increase the amount of extracted DNA [18]. Thus, the application of separate ultrasound procedure in the InnowaveDX assay may be another explanation for its low LOD for MTB detection.

The increased sensitivity of InnowaveDX would provide potential benefits to detect more active TB patients with low bacterial load when InnowaveDX was assessed on 951 sputum samples from patients with symptoms suggestive of TB. Sixty-one additional clinically diagnosed patients were correctly identified by InnowaveDX, indicating its superior detection capability for paucibacillary specimens. By contrast, we also found that the InnowaveDX assay failed to detect a small proportion of Xpert-positive cases, even smear-positive cases. These conflicting results may be explained by its notoriously non-homogenous characteristic [19], and the nonuniform distribution of tubercle bacilli and PCR inhibitors are be associated with the false-negative results by the InnowaveDX. In view of this point, we propose schemes used for potentially aiding the diagnosis of TB. First, the homogenization of sputum before laboratory examination is essential to disrupt uneven distribution of bacilli, enhance the release of bacilli in solution, and decrease the likelihood of discordant results by different assays. Second, the combined application of bacteriological culture and ultrasensitive molecular techniques may serve as a useful strategy to overcome drawbacks associated with the non-homogeneous sputum.

Although a heightened sensitivity to bring benefits to individuals at risk for false negatives, it may come at the cost of decreased specificity, thereby significantly influencing the likelihood of false positives. In populations with low bacterial load, the positive predictive value of the InnowaveDX assay will be even lower than what we have reported, such as those living with HIV and those that were smear negative. The increase in sensitivity also raises concern for an elevated risk of false-positive results due to sample cross contamination. The pre-PCR processing of samples is prone to generate a significant amount of aerosols [20], especially in laboratories that have numerous samples containing tubercle bacilli. This may be a major case for false positive cases by InnowaveDX in the present study. Importantly, increased false-positive results have been reported when the ultrasensitive molecular assay, such as Xpert, were used in individuals with recent episode of tuberculosis. In this regard, InnowaveDX is problematic when used for diagnosis of patients following treatment given the fact that they cannot distinguish between alive and dead bacilli, highlighting that more attention should be paid to indications and interpretation of results.

InnowaveDX also enables detection of mutations within rifampicin resistance determining region. However, the LOD for detecting MTB was at least l.5 log better for detecting wild type and mutants in *rpoB* target, because it exists in a single copy in bacterial genomes. Notably, our data have confirmed that the discordant RIF susceptibility results were more frequently observed in samples with very low bacterial load. Consistent with our study, a retrospective study by Huo and coresearchers demonstrated that one fifth of isolates with discordant results between Xpert and phenotypic DST lacked *rpoB* mutations, a result majorly noted in specimens with very low bacterial load [21]. Taken together, more caution should be exercised regarding interpretation of RIF susceptibility when evaluating the cases with low bacterial load in view of the increased risk for false-positive RIF-resistance.

Although the Foundation for Innovative and New Diagnostics (FIND) negotiated a discounted price of Xpert for public section in high TB-burden and developing countries[22], this negotiated price was not applicable to China. The high cost of Xpert cartridge (∼$65) is seen as a key hurdle to implementation in this country. In this respect, the InnowaveDX assay has a low cost per test (∼$10), about a seventh of the price of Xpert. Additionally, InnowaveDX provides comparable turnaround time to identify the presence of MTB as Xpert, and could handle a higher batch capacity (a batch of 48 samples). All these advantages make InnowaveDX an alternative for being routinely practiced in the resource-limited settings.

We also acknowledged several obvious limitations to this study. First, a previous analytical study on Xpert Ultra revealed that this assay had a better TB detection capability [13]. However, we did not compare its diagnostic accuracy with InnowaveDX in clinical practice. Second, InnowaveDX could only identify RIF resistance rather than INH resistance. Although RIF resistance has been used as a surrogate marker for MDR-TB [23], there is a growing concern for the high proportion of RIF monoresistance in China [24], emphasizing the need to simultaneously detect resistance to RIF and INH. Third, the inclusion of multicopy IS6110 leads the increase in sensitivity of InnowaveDX; however, the IS6110 copy numbers exhibited remarkable difference across MTB strains, even lack of a copy of IS6110 in some clinical strains [25]. Thus, the sensitivity decrements for InnowaveDX may be great in the settings where these IS6110-depleted strains are prevalent. Fourth, despite yielding additional value of diagnosing culture-negative TB cases, we noticed that the sensitivity of InnowaveDX was 95% for smear-positive patients, slightly lower than the WHO target product profile for this population (98%) [26]. A potential explanation for this result may be related to the nonhomogeneous nature of sputum. Fifth, the *in vitro* DST results were only used to interpret discordance between two molecular assays because a proportion of confirmed TB patients were culture-negative, further hampering phenotypic DST analysis. Finally, mutations in the *rpoB* gene were not determined by DNA sequencing. Further analysis is warranted to confirm the observation regarding the accuracy of molecular diagnostics applied in specimens with low bacterial loads.

To conclude, we have developed a novel molecular diagnostic with promising detection capabilities of TB and RIF susceptibility. In addition, the discordant RIF susceptibility results between InnowaveDX and Xpert are more frequently observed in samples with very low bacterial load, indicating that more caution should be exercised regarding interpretation of RIF susceptibility when evaluating these cases.

## Supplementary Material

Supplemental Material

Supplemental Material
