# Supplementary material for: Accuracy of the InnowaveDX MTB/RIF test for detection of Mycobacterium tuberculosis and rifampicin resistance: a prospective multicentre study
Source: Emerg Microbes Infect. 2023 Jan 2;12(1):2151382. doi: 10.1080/22221751.2022.2151382 (PMC9815255; doi:10.1080/22221751.2022.2151382)
Supplement: Supplemental Material [file TEMI_A_2151382_SM7948.docx]

Table S1 14 species of nontuberculous mycobacteria (NTM) and other bacteria used for specificity analysis of InnowaveDX

| Species | Name | Strain number^a^ |
| --- | --- | --- |
| NTM | *Mycobacterium avium* | CMCC95001 |
|  | *Mycobacterium terrae* | CMCC95005 |
|  | *Mycobacterium shimoidei* | CMCC95008 |
|  | *Mycobacterium kansassi* | CMCC95103 |
|  | *Mycobacterium asiaticum* | CMCC95016 |
|  | *Mycobacterium scrofulaceum* | CMCC95017 |
|  | *Mycobacterium gordonae* | CMCC95018 |
|  | *Mycobacterium chelonae* | CMCC95021 |
|  | *Mycobacterium fortuitum* | CMCC95022 |
|  | *Mycobacterium phlei* | CMCC95024 |
| Other | *Nocardia brasiliensis* | CGMCC4.1128 |
|  | *Corynebacterium pekinense* | CGMCC1.0295 |
|  | *Pneumococci* | CMCC31130 |
|  | *Legionella pneumophila* | CDCBJ-2 |

^a^The strains of *Mycobacterium avium, Mycobacterium terrae, Mycobacterium shimoidei, Mycobacterium kansassi, Mycobacterium asiaticum, Mycobacterium scrofulaceum, Mycobacterium gordonae, Mycobacterium chelonae, Mycobacterium fortuitum, Mycobacterium phlei* and *Pneumococci* were provided by National Center for Medical Culture Collections of China (CMCC); the strains of *Nocardia brasiliensis* and *Corynebacterium pekinense* were provided by China General Microbiological Culture Collection Center (CGMCC);the strain of *Legionella pneumophila* was provided by Chinsed Center for Disease Control and Prevention (CDC).
