# Supplementary material for: Accuracy of the InnowaveDX MTB/RIF test for detection of Mycobacterium tuberculosis and rifampicin resistance: a prospective multicentre study
Source: Emerg Microbes Infect. 2023 Jan 2;12(1):2151382. doi: 10.1080/22221751.2022.2151382 (PMC9815255; doi:10.1080/22221751.2022.2151382)
Supplement: Supplemental Material [file TEMI_A_2151382_SM7946.docx]

Table S2 Demographic characteristics of enrolled suspected TB patients

| Characteristics | No. of patients（N=951） |
| --- | --- |
| Median age (range) | 52.3(3.0-92.0) |
| Male sex- no. (%) | 672(70.7%) |
| Region (%) |  |
| Beijing | 97(10.2%) |
| Guangzhou | 205(21.6%) |
| Hangzhou | 104(10.9%) |
| Jinan | 148(15.6%) |
| Xian | 157(16.5%) |
| Xinjiang | 100(10.5%) |
| Changsha | 140(14.7%) |
| Comorbidity (%) |  |
| Diabetes | 4(0.4%) |
| Liver diseases | 4(0.4%) |

Abbreviations: TB=tuberculosis.
